# Altered White Matter Architecture in *BDNF* Met Carriers

**DOI:** 10.1371/journal.pone.0069290

**Published:** 2013-07-31

**Authors:** Erik Ziegler, Ariane Foret, Laura Mascetti, Vincenzo Muto, Anahita Le Bourdiec-Shaffii, Johan Stender, Evelyne Balteau, Vinciane Dideberg, Vincent Bours, Pierre Maquet, Christophe Phillips

**Affiliations:** 1 Cyclotron Research Centre, Université de Liège, Liège, Belgium; 2 Department of Human Genetics, CHU Sart Tilman, Liège, Belgium; 3 Department of Neurology, CHU Liège, Liège, Belgium; 4 Department of Electrical Engineering and Computer Science, University of Liège, Liège, Belgium; Institut National de la Santé et de la Recherche Médicale (INSERM U901), France

## Abstract

Brain-derived neurotrophic factor (BDNF) modulates the pruning of synaptically silent axonal arbors. The Met allele of the *BDNF* gene is associated with a reduction in the neurotrophin's activity-dependent release. We used diffusion-weighted imaging to construct structural brain networks for 36 healthy subjects with known *BDNF* genotypes. Through permutation testing we discovered clear differences in connection strength between subjects carrying the Met allele and those homozygotic for the Val allele. We trained a Gaussian process classifier capable of identifying the subjects' allelic group with 86% accuracy and high predictive value. In Met carriers structural connectivity was greatly increased throughout the forebrain, particularly in connections corresponding to the anterior and superior corona radiata as well as corticothalamic and corticospinal projections from the sensorimotor, premotor, and prefrontal portions of the internal capsule. Interhemispheric connectivity was also increased via the corpus callosum and anterior commissure, and extremely high connectivity values were found between inferior medial frontal polar regions via the anterior forceps. We propose that the decreased availability of BDNF leads to deficits in axonal maintenance in carriers of the Met allele, and that this produces mesoscale changes in white matter architecture.

## Introduction

Secretion of brain-derived neurotrophic factor is essential for synaptic plasticity in the central nervous system during neurodevelopment [1], as well as in mature brains, in which it promotes long-term potentiation and the formation of long-term memory [2], [3]. A common human non-synonymous single-nucleotide polymorphism in the *BDNF* gene (Val66Met, rs6265) decreases activity-dependent BDNF release in neurons transfected with the human A allele (Met-*BDNF*) [4]. It is also associated with variation in human memory [5], [6], and with several neurological and psychiatric disorders [7]. We reasoned that the persistent differential activity-dependent BDNF release implied by this polymorphism should also be associated with differences in adult brain structure. Accordingly, the polymorphism affects the anatomy of the hippocampus and prefrontal cortex [8]. In this study we examine structural connectivity in the brains of normal human participants stratified according to *BDNF* genotypic group.

Indeed, for any equivalent set of connections, there is substantial variability in the density of cortical fibers between individuals of the same species [9]. This variability is in part genetically determined. Functional MRI in monozygotic and dizygotic twins has shown that 60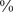 or more of the inter-subject variance in transmission efficiency of cortical networks can be attributed to genetic effects [10]. However, the mechanisms by which this genetic influence impacts human brain connectivity are not yet determined. Comparison of groups by *BDNF* genotype may be useful for assessing the impact of activity-dependent processes on brain connectivity.

Here, we originally hypothesized that there would be decreased structural connectivity in Met carriers corresponding to the reduced availability of the neurotrophin. We examined a healthy young population with diffusion-weighted MR imaging, reconstructed their white matter tracts with probabilistic tractography, and examined the effect of carrying the *BDNF* Met allele at the connectome level. Contrary to our hypothesis, we found a marked increase in connectivity strength as well as altered track topology for Met carriers.

## Results

### Population

In our cohort (n = 134), the studied non-synonymous coding single-nucleotide polymorphism (rs6265) was in Hardy-Weinberg equilibrium (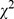 = 3.25, p = 0.07) with genotypic frequency of 0.6 (G/G), 0.31 (G/A) and 0.09 (A/A). The final study population comprised 36 healthy subjects aged 18–25. Fifteen (9 male) were identified as carrying the Met allele. The remaining 21 (9 male) were homozygotes for the Val allele and were referred to as the Val/Val group. The groups did not vary significantly in IQ or age, nor did their scores differ for a battery of psychological tests (**Table S1 in File S1**).

### Network-based statistics

**Figure 1:**
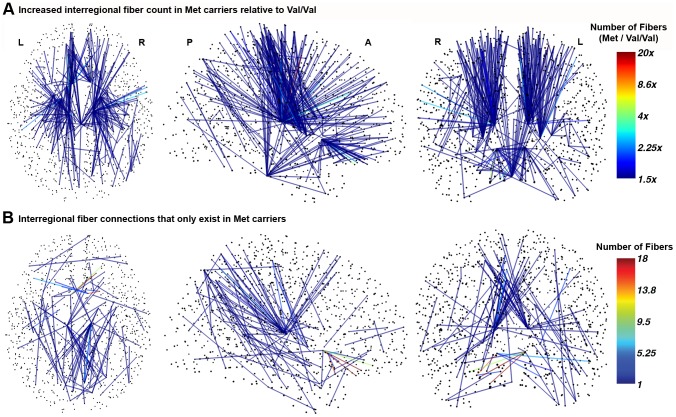
Significantly increased regional connectivity and topological changes in Met carriers. (**a**) Track count increase for each connected edge in Met carriers (n = 15) versus Val/Val subjects (n = 21). (**b**) Region-to-region track pathways that are present only in the Met carriers.

In our networks, with 1015 nodes and an average of 66,456 edges, we identified 387 connections in which the number of connecting tracks was significantly greater in carriers of the Met allele than in the Val homozygotes (p = 0.0122, permutation testing). The relative connection strengths for these edges are shown in **Fig. 1a**.

For Met carriers the strength at these edges was found to range between 1.75 and 48 times their strength in Val/Val. Of these edges, 41 (11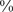) were found to have between 75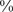 and 200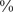 more tracks in Met carriers than in Val homozygotes. Met carriers had 200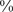 to 400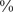 more tracks in 123 (32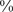) of the edges, 400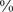 to 900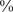 more tracks in 104 edges (27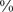), and even greater factors in the remaining 23 edges (6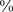). The affected edges were largely central connections and were not short or uncommon fiber pathways.

Roughly one quarter (96) of the edges that were identified were not present in any of the Val/Val subjects (i.e. the mean value in Met carriers was significantly greater than the value of zero, found in Val homozygotes). **Fig. 1b** shows the mean number of tracks for the 96 edges that were only present in the Met carriers. The connections unique to Met carriers appeared consistently across the group. We did not find any edges with significantly lower strength in Met carriers. The identified connectivity changes are unlikely to represent false positives because of the stringent non-parametric statistical method [11]. Moreover, the reported differences were specific to the *BDNF* polymorphism; subjects were also divided by gender (18 F, 18M), and by their adenosine deaminase (ADA) genotype (17 GA, 19 GG), and no significant results were obtained.

Global network metrics (graph density, number of connected components, transitivity) showed no variation between groups. Local nodal metrics (degree, clustering coefficient, number of triangles, [closeness, betweenness, degree] centrality, highest *k*-core number) were averaged for each participant and also did not vary. Wiring cost and network efficiency, compared both for the whole network as well as for only corticocortical connections, were unaffected by *BDNF* genotype. The total number of tracks per connectome, out of the generated 300,000 per subject, did not differ. The lack of significant variation in any of the network metrics is understandable because the total number of altered edges (387) is less than 1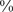 of the mean number of edges (66,456) per network.

### Classifier performance

**Figure 2:**
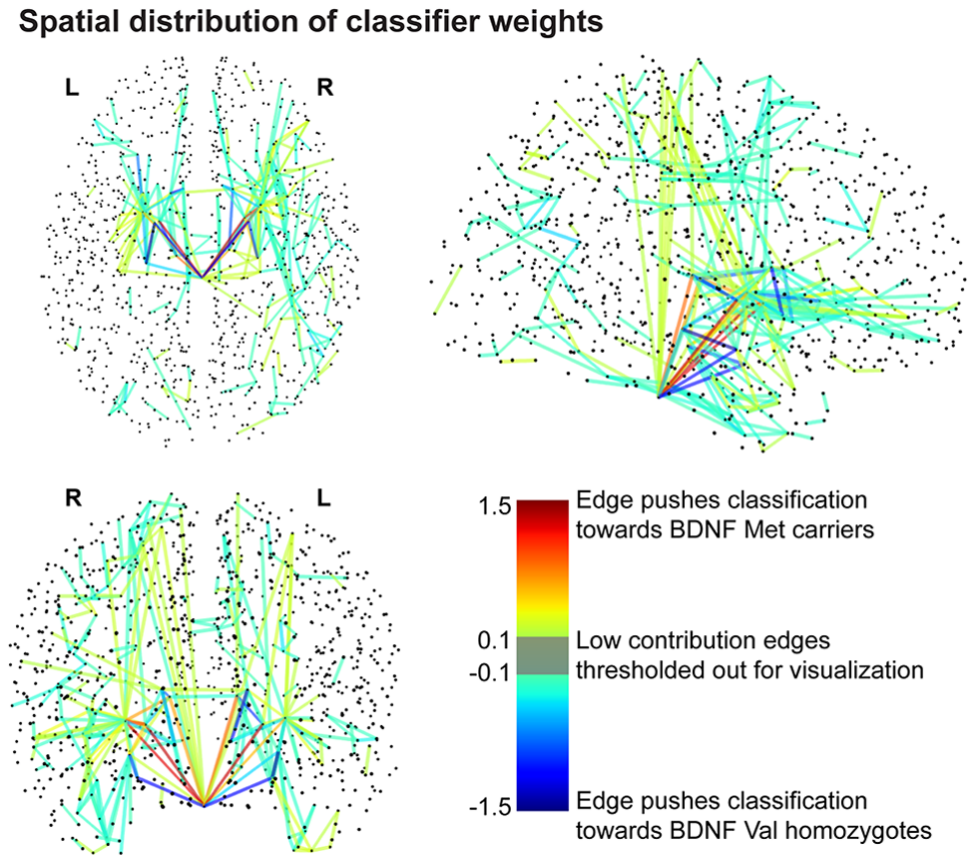
Classifier weight distribution. The weights obtained by the classifier have been plotted as network edges in order to show their spatial distribution. The thresholding procedure removed 99.75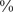 of the edges for clarity. The remaining connections represent 21.69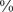 of the absolute weight.

The classifier was able to discriminate between Val homozygotes and Met carriers with 86.1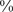 global accuracy. The predictive value for the Val/Val and Met carrier groups were 94.4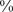 (p = 0.001) and 77.8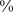 (p = 0.003), respectively. In **Figure 2** the weights obtained by the classifier are visualized as edges in the brain network. For the classifier trained to identify gender, the global accuracy reached 63.9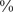 (n.s.). Identifying the subjects' adenosine deaminase (ADA) genotype was only possible with an accuracy of 58.3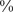 (n.s.).

### Tractographic basis

**Figure 3:**
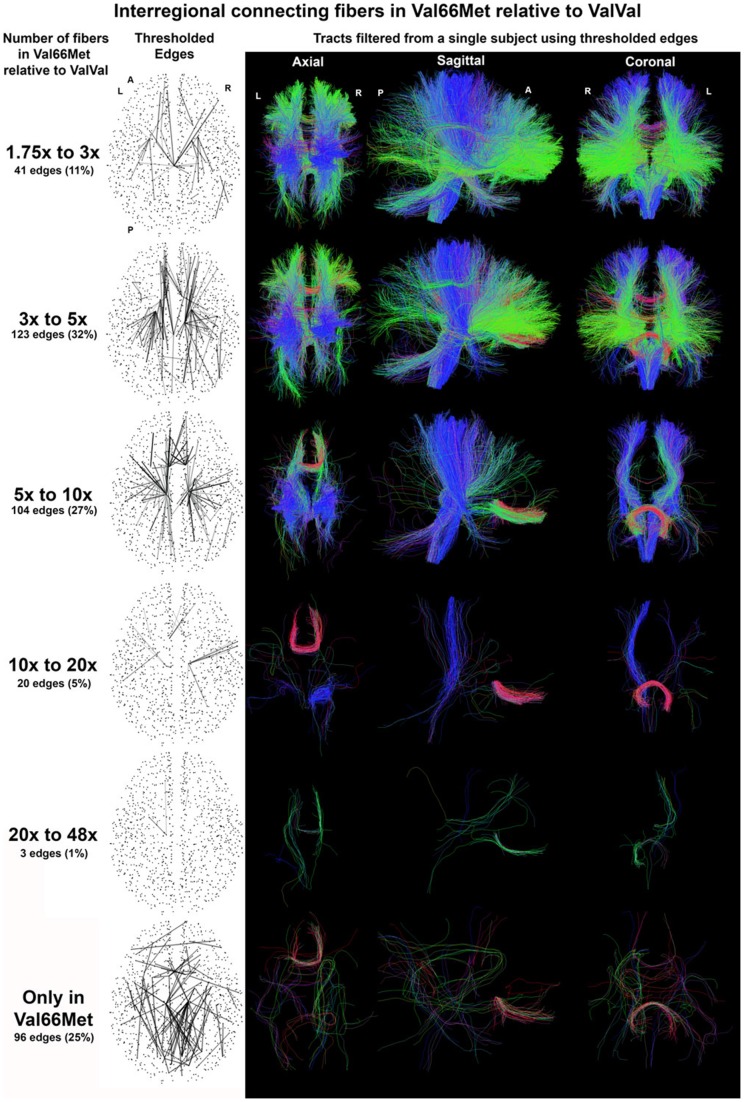
Connecting tracks in Met carriers. The average number of tracks connecting two regions (i.e. the edge weight) in Met carriers was found to range from 1.75 to 48x the value found in the Val/Val group. The range was so broad that it had to be analyzed in separate stages. Fibers shown are filtered from a single Met carrier.

Structural connectivity in Met allele carriers was found to be higher throughout the forebrain (**Figure 3**). Large increases were found in connections corresponding to the anterior and superior corona radiata, including the corticothalamic and corticospinal projections from the sensorimotor, premotor, and prefrontal portions of the internal capsule. General interhemispheric connectivity was increased via the corpus callosum and anterior commissure. Extremely high connectivity values were found between inferior medial frontal polar regions via the anterior forceps. The Met carriers also presented novel connections within the cingulum, corpus callosum, and anterior forceps, which were not found in the Val homozygotes.

## Discussion

Using high-resolution connectome mapping, we observe significant differences in structural brain connectivity between samples of normal young healthy human volunteers recruited based on the Met allele of the *BDNF* gene. These differences appear to involve specific fiber tracts; although widespread, they do not modify connectome parameters computed over the whole brain. They also appear specific to this allele; no such difference could be found for the polymorphism in the adenosine deaminase gene, or even for gender. We further demonstrate that this structural information can be used, with a reasonably high accuracy, to identify the *BDNF* genotype of an individual from his structural brain wiring.

In many regions the number of connecting tracks in Met carriers is increased by a factor of 3 or more. These are substantial changes at a mesoscopic anatomical level that are in line with previous findings by other groups. One large study examined fractional anisotropy (FA) – a measure of the restrictedness of random motion in water molecules – in 455 subjects and reported higher values, in some areas by up to 15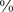, in Met carriers [12]. A larger number of fibers oriented in the same direction would necessarily increase local anisotropy. Our findings confirm and extend their findings by specifying the nature and topology of these differences. It is not white matter integrity that is altered between Met carriers and Val homozygotes, but rather the strength and architecture of their white matter tracts. The connections with increased strength in Met carriers predominantly involve the thalamus and brainstem, the sensorimotor areas of parietal and frontal cortex, and the ventral medial prefrontal cortex. The occipital, posterior parietal, and temporal areas also appear to differ between allelic groups to a lesser extent.

It must be stated that the results obtained here are dependent on the regional parcellation of the structural brain images. Previous studies have shown that the choice of region size and number greatly impacts the resulting network metrics [13], [14]. In this work we chose to use a previously published and open-source parcellation scheme that depends on automated atlas-based segmentation [14]–[17].

Intriguingly, these anatomical changes do not translate into improved performance in either of our populations. Indeed, by design, our samples were matched for various demographic variables including IQ, age, and education level. One possible explanation for this phenomenon is that the increased connection strengths are due to redundant connections that are not essential to sustain the speed or efficiency of information processing.

The mechanisms causing these alterations cannot be derived from the current data. However, in addition to its involvement in long-term potentiation and synaptic plasticity [2], BDNF has also been implicated in axonal pruning and maintenance. BDNF is released from stimulated “winning” neurites and binds to the p75NTR receptor on nearby “losing” terminals, triggering the elimination of synaptically silent axonal terminal arbors [18], [19]. It is tempting to suggest that the reduction in activity-dependent BDNF secretion accounts for the observed changes in white matter architecture. If indeed silent axons are relatively less likely to be pruned due to reduced BDNF secretion in Met carriers, brain connectivity might eventually be less profoundly shaped by experience than in homozygous Val individuals, without any conspicuous behavioral consequences.

In keeping with this hypothesis, brain maturation from childhood to adolescence is a nonlinear regionally selective process [20]. Gray matter loss is abundant, as is axonal myelination, and both continue until early adulthood. Consistent with our findings, grey matter volume in adults was shown to be lower in Met carriers in both the lateral frontal cortices and hippocampi [8]. Moreover, these differences were deemed independent of age (18 to 60) and gender, which suggests that the morphological changes are occurring prior to adulthood. It is possible that the increase we identify in connecting tracks is a result of deficits in axonal maintenance during adolescence, a key period of synaptic revision. When tested at age 11, children in a longitudinal study showed no differences in verbal reasoning that could be associated with their *BDNF* genotype. When the same cohort was tested again, the elderly Met homozygotes outperformed heterozygotes as well as their homozygous Val counterparts in both verbal and non-verbal reasoning [21]. It has also been reported that Met carriers show enhanced task-switching abilities during old age [22]. These convergent findings support the idea that the Met allele protects against age-related detriments in brain function, possibly by providing redundant or degenerate connectivity.

Finally, although we matched our population samples with great care and conducted conservative statistical analyses, our study is not immune from random sampling biases. The absence of significant results concerning ADA polymorphism and gender indicate that the reported effects are specific to *BDNF* polymorphism. However, contradictory results have been reported about the effect of *BDNF* polymorphism on cognitive performance and disease susceptibility [23], potentially caused by genetic interactions and global haplotypic diversity [24]. It is important to note that the Val66Met polymorphism has a wide variation in prevalence worldwide. Its frequency ranges from 0.55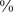 in Sub-Saharan Africa, to 19.9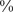 in Europe, and 43.6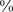 in Asia [24]. Studies including subjects from different populations should take care to consider their genetic backgrounds.

Future research should confirm these findings in healthy populations of both young and old subjects, as well as during the development period from childhood to adolescence. Longitudinal neuroimaging data would clarify BDNF's effect on brain development and connectivity, and larger populations may help identify whether these changes can be fully attributed to the Met allele. It also remains to be seen if these alterations are more or less profound in Met homozygotes or in subjects with the Val66Met polymorphism. The prevalence of the Met allele [24], [25] suggests that it confers some evolutionary advantages. It may be that these advantages, developed during preadolescence, are only manifested in old age.

## Methods

### Ethics Statement

Volunteers were recruited through advertisement on the University intranet. They gave their written informed consent to participate in the study, which was approved by the Ethics Committee of the Faculty of Medicine at the University of Liège.

### Population

Participants were young (18–25 years old), healthy, and lean (Body Mass Index 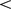
26). They were all right-handed, as determined by the Edinburgh Inventory [26]. None complained about sleep disturbances, and this was reflected by the Pittsburgh Sleep Quality Index (PSQI score 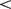
6) [27]. Extreme chronotypes, according to the Horne and Ostberg morningness-eveningness questionnaire, were excluded (scores 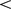
31 or 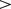
69) [28]. Their sleep midpoint on free days was required to be between 3 and 5.99 as indicated by the Munich Chronotype Questionnaire [29]. They all scored in the normal range (0–9) on the Epworth Sleepiness Scale [30]. The absence of medical, traumatic, psychiatric, and sleep disorders was established through a semi-structured interview.

All participants had normal scores on the 21-item Beck Anxiety Inventory (score 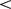
11) and the 21-item Beck Depression Inventory-II (score 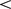
14) [31], [32]. They were non-smokers and moderate caffeine and alcohol consumers. None were on medication other than oral contraceptives. No caffeine was allowed during the experiment.

Volunteers complying with these criteria were invited to perform Raven's Progressive Matrices and a blood sample was obtained for *BDNF* genotyping [33]. Participants were eventually selected based on their *BDNF* genotype. Allelic groups were formed with participants that were matched according to gender, age, education level, chronotype, PSQI score and IQ (**Table S1 in File S1**). Subjects received financial compensation for their blood test and participation in the study.

### Genotyping

Genomic DNA was extracted from blood samples using a MagNA Pure LC Instrument. The DNA sequence of interest was amplified by Polymerase Chain Reaction in a final volume of 50 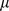
l containing 0.6 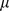
M of each primer (Thermo Scientific, Germany), 0.5 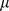
l Faststart Taq DNA Polymerase (Roche Diagnostics, Germany), 0.8 mM of each deoxynucleotide triphosphate (Roche Diagnostics Germany) and 20 ng of genomic DNA. After 10 min of denaturation at 95°C, samples underwent 35 cycles consisting of denaturation (95°C, 30 sec), annealing (60°C, 40 sec), and extension (72°C, 30 sec), followed by a final extension of 7 min at 72°C. The amplified DNA product was then subjected to pyrosequencing (Pyromark Q96 Vacuum Workstation, PSQ 96MA, Pyromark Gold Q96 Reagents, Qiagen, Germany). The sequences of the primers are available upon request.

### Data Acquisition

Data was acquired on a 3 T head-only scanner (Magnetom Allegra, Siemens Medical Solutions, Erlangen, Germany) operated with the standard transmit-receive quadrature head coil. A high-resolution T1-weighted image was acquired for each subject (3D modified driven equilibrium Fourier transform, repetition time  = 7.92 ms, echo time  = 2.4 ms, inversion time  = 910 ms, flip angle  = 15°, field of view  = 256×224×176 mm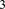, 1 mm isotropic spatial resolution). Seven unweighted (b = 0) volumes were acquired followed by a set of diffusion-weighted (b = 1000) images using 61 non-collinear directional gradients.

### Processing & Analysis

The processing workflow was developed in Python and imports modules from the Nipype project [34]. The pipelines used for both single subjects and groups have been detailed as part of the online Nipype documentation in order to improve transparency and promote reproducibility. Every piece of software (CMTK, ConnectomeViewer, Dipy, Freesurfer, FSL, Nipype, Nibabel, MRtrix) used to process data in this paper is currently operating under an open-source license. The process began by segmenting the structural MR images using the automated labeling of Freesurfer [15]. Segmented structural images were then further parcellated using the Lausanne2008 atlas for a total of 1015 regions of interest (ROIs) [14]. Diffusion-weighted images were corrected for image distortions arising from eddy currents using linear coregistration functions from the FMRIB Software Library (FSL) [35]. Fractional anisotropy maps were generated, and a small number of single-fiber (high FA) voxels were used to estimate the spherical harmonic coefficients of the response function from the diffusion-weighted images [36], [37]. Using non-negativity constrained spherical deconvolution, fiber orientation distribution (FOD) functions were obtained at each voxel. For our dataset with 61 directions, we used the maximum allowable harmonic order of 8 for both the response estimation and spherical deconvolution steps. Probabilistic tractography was performed throughout the whole brain using seeds from subject-specific white-matter masks and a predefined number of tracts. Fiber tracking settings were as follows: number of tracks  = 300,000, FA/FOD amplitude cutoff for terminating tracks  = 0.1, minimum track length  = 10 mm, maximum track length  = 200 mm, minimum radius of curvature  = 1 mm, tracking algorithm step size  = 0.2 mm. Using tools from Dipy (Diffusion in Python, http://nipy.sourceforge.net/dipy/), the tracks were affine-transformed into the subject's structural space. This procedure circumvents the common problem of having to downsample ROI image files – defined in structural space – so that they can be used in diffusion space for connectivity mapping, and therefore leads to more accurate connectomes. Connectome mapping was performed by considering every contact point between each tract and the outlined regions of interest. Unlike in some past papers (e.g. [14], [16]) which considered only fiber start and endpoints, we incremented our connectivity matrix every time a single fiber traversed between any two regions. This leads to a far denser network than we have seen before, presumably with more accurate network properties. The number of tracked fibers which remained in each subject's connectome was also recorded.

This method of connection mapping may need further optimisation, however, as it can potentially be linking gray matter cortical regions through unreliably tracked fibers. This is something that may be avoidable by placing limits on the propagation parameters, or with anatomically or otherwise constrained tractography approaches [38], [39]. The benefits and drawbacks of different mapping techniques should be explored by future studies.

Numerous network metrics were obtained for each connectome and compared at the group level. At the nodal level we calculated the degree, clustering coefficient, and number of triangles, as well as three measures of centrality (closeness, betweenness, degree), and the highest *k* (i.e. degree) value for each *k*-core the node is encompassed by. For the network as a whole we computed the average shortest path length (i.e. the inverse of efficiency), the wiring cost (using Euclidean distance between nodes), the graph density, the number of connected components, and the graph's transitivity [40]. For a complete description of all of these metrics, the reader is referred to the Python package NetworkX [41]. Tract and network visualization were performed in TrackVis (Ruopeng Wang, Van J. Wedeen, TrackVis.org, Martinos Center for Biomedical Imaging, Massachusetts General Hospital), MRtrix, and ConnectomeViewer [42]. **Figure S2** provides, for visualization, the orientation distribution functions and generated fiber tracts for a midbrain coronal slice of a single subject. In **Figure S3**, the structural connectome and T1-weighted image are shown for the same subject, and thresholded across two distinct fiber-count ranges, so that both the core and the density of the network can be seen.

### Statistical Analysis

The Network-based Statistic (NBS) was used to identify differences between *BDNF* allelic groups (**Fig. S1a**) [11]. For each permutation the t-values at each edge were thresholded above a value of 3. The supra-threshold components were then compared against the generated null distribution. The null distribution for each test was produced by permuting members of each population 5000 times and estimating the maximal component size.

A table describing a representative subject's connection matrix and edge weights is given in **Table S2 in File S1**. Since the networks in this study have a high number of regions, and we have performed whole-brain connectome mapping with a relatively low number of fibers, a large proportion of our network's edges have low fiber counts. This may be problematic for statistical testing with the NBS because these small-integer populations do not provide wide ranges for edge weights and can result in inaccurate t-values. In future, it may be prudent to generate a larger number of streamlines, reduce the number of nodes in the network, or restrict analysis to specific parts of the brain. Practically, it can be computationally intensive to deal with large streamline datasets and networks with high numbers of nodes. The trade-off between resolution and resources is something that must be decided by the researcher with the focus of the study in mind.

In **Fig. S1b** we projected the observed NBS component onto the tractography of a single subject. This projection is, in effect, a type of reverse connectome-mapping. Given the connectivity network, we filtered the set of tracts to show only those that traverse between regions with edges in the network. Global graph metrics, psychological metrics, and the total number of fibers per connectome, were compared directly between allelic groups via Student's t-test. Nodal measures were averaged for each subject and analyzed in the same manner. All distributions were plotted as combined histogram/kernel-density maps to evaluate gaussianity prior to statistical analysis. Apart from the results given by the network-based statistic, no significant differences were identified between the two genotypic groups for any of the graph-level measures. No significant differences were observed between allelic groups in any of the psychological metrics.

### Classification

The multivariate statistical properties of our data were studied with a linear Gaussian Process Classification method [43] as interfaced by PRoNTo (Pattern Recognition for Neuroimaging Toolbox, http://www.mlnl.cs.ucl.ac.uk/pronto) [44]. The classifier was given the fiber-count connection matrices for each subject and their true classes (e.g. Met carrier, Female). No network metrics, topology, or spatial information was provided to the classifier. The accuracy and generalisability of the classification were assessed with a leave-one-out cross-validation procedure: one subject is left out at a time, the classifier is trained on the remaining data, and the true and predicted (by the trained classifier) classes of the left-out subject are compared. With this linear kernel method weights were also obtained indicating the contribution to the classification output (in favor of either genotypic group) of each edge in the network. The same method was employed to discriminate features related to the subjects' gender and genotype for the ADA gene. For the purposes of visualization, we thresholded the edges in **Figure 2**. The removed portion of the classification weights can be found in **Fig. S4**. Example calculations for the percent classification weight represented by the remaining edges can be found in the Supplementary Information in File S1.

## Supporting Information

Figure S1 **Edge weights are stronger in Met carriers.** (**a**) In the structural component pictured each inter-regional connection has a significantly higher number of tracks for Met carriers. (**b**) The tracks shown are produced by filtering a single subject's tracts using the connections from the network shown in (**a**).
(TIF)

Figure S2 **Tracks and Orientation Distribution Functions for a single subject.** Combined figure for visualizing the results of the spherical deconvolution and probabilistic fiber tractography steps in the processing pipeline.
(TIF)

Figure S3 **Structural connectome for a single subject.** Structural connectivity network built from the Lausanne 2008 regional atlas – with each region displayed as a node – and a set of 300,000 fiber tracks. Colored edge weights represent the number of tracks that provide any connection between any pair of regions. The figure is divided into ranges of edge weights for optimal visualization of the (**a**) high-valued structural core and the (**b**) low-valued associative connections.
(TIF)

Figure S4
**Detailed dissection of the classification weights.** (**a**) The complement of Figure 2 from the main text. This network details the edges that were filtered in the main text figure, and shows 99.75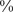 of the edges, which represent only 78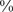 of the total weight. (**b**) A set of very low contribution edges between genotypic groups. These very low-valued edges are difficult to interpret. (**c**) The highest valued edges that were thresholded out of Figure 2 in the main text. A pattern of posterior parietal and medial frontal connectivity can be inferred in the Met carriers, but the abundance of edges is still complex to visualize.(TIF)

File S1 **Table S1– Psychological questionnaire results.** Values reflect mean ± standard deviation. **Table S2– Connectome edge weights.** This table details a single random (Val) subject's network edges. The vast majority of the edges had weights below a fiber count of 100.
(PDF)

## References

[1] Huang EJ, Reichardt LF (2001) Neurotrophins: roles in neuronal development and function. Annu Rev Neurosci 24: 677–736. 11520916 10.1146/annurev.neuro.24.1.677 PMC2758233

[2] Patterson SL, Abel T, Deuel TA, Martin KC, Rose JC, et al (1996) Recombinant BDNF rescues deficits in basal synaptic transmission and hippocampal LTP in BDNF knockout mice. Neuron 16: 1137–1145. 8663990 10.1016/s0896-6273(00)80140-3

[3] Bekinschtein P, Cammarota M, Katche C, Slipczuk L, Rossato JI, et al (2008) BDNF is essential to promote persistence of long-term memory storage. Proc Natl Acad Sci U S A 105: 2711–2716. 18263738 10.1073/pnas.0711863105 PMC2268201

[4] Egan MF, Kojima M, Callicott JH, Goldberg TE, Kolachana BS, et al (2003) The BDNF val66met polymorphism affects activity-dependent secretion of BDNF and human memory and hippocampal function. Cell 112: 257–269. 12553913 10.1016/s0092-8674(03)00035-7

[5] Cheeran B, Talelli P, Mori F, Koch G, Suppa A, et al (2008) A common polymorphism in the brain-derived neurotrophic factor gene (bdnf) modulates human cortical plasticity and the response to rtms. J Physiol 586: 5717–5725. 18845611 10.1113/jphysiol.2008.159905 PMC2655403

[6] Goldberg TE, Iudicello J, Russo C, Elvevg B, Straub R, et al (2008) Bdnf val66met polymorphism significantly affects d' in verbal recognition memory at short and long delays. Biol Psychol 77: 20–24. 17988784 10.1016/j.biopsycho.2007.08.009

[7] Hong CJ, Liou YJ, Tsai SJ (2011) Effects of BDNF polymorphisms on brain function and behavior in health and disease. Brain Res Bull 86: 287–297. 21924328 10.1016/j.brainresbull.2011.08.019

[8] Pezawas L, Verchinski BA, Mattay VS, Callicott JH, Kolachana BS, et al (2004) The brain-derived neurotrophic factor val66met polymorphism and variation in human cortical morphology.J Neurosci. 24: 10099–10102. 10.1523/JNEUROSCI.2680-04.2004 PMC6730170 15537879

[9] Hilgetag CC, Grant S (2000) Uniformity, specificity and variability of corticocortical connectivity.Philos Trans R Soc Lond B Biol Sci. 355: 7–20. 10.1098/rstb.2000.0546 PMC1692717 10703041

[10] Fornito A, Zalesky A, Bassett DS, Meunier D, Ellison-Wright I, et al (2011) Genetic influences on cost-efficient organization of human cortical functional networks. J Neurosci 31: 3261–3270. 21368038 10.1523/JNEUROSCI.4858-10.2011 PMC6623940

[11] Zalesky A, Fornito A, Bullmore ET (2010) Network-based statistic: identifying differences in brain networks. Neuroimage 53: 1197–1207. 20600983 10.1016/j.neuroimage.2010.06.041

[12] Chiang MC, Barysheva M, Toga AW, Medland SE, Hansell NK, et al (2011) BDNF gene effects on brain circuitry replicated in 455 twins. Neuroimage 55: 448–454. 21195196 10.1016/j.neuroimage.2010.12.053 PMC3192852

[13] Zalesky A, Fornito A, Harding IH, Cocchi L, Ycel M, et al (2010) Whole-brain anatomical networks: does the choice of nodes matter? Neuroimage 50: 970–983. 20035887 10.1016/j.neuroimage.2009.12.027

[14] Cammoun L, Gigandet X, Meskaldji D, Thiran JP, Sporns O, et al (2012) Mapping the human connectome at multiple scales with diffusion spectrum mri. J Neurosci Methods 203: 386–397. 22001222 10.1016/j.jneumeth.2011.09.031

[15] Desikan RS, Sgonne F, Fischl B, Quinn BT, Dickerson BC, et al (2006) An automated labeling system for subdividing the human cerebral cortex on MRI scans into gyral based regions of interest. Neuroimage 31: 968–980. 16530430 10.1016/j.neuroimage.2006.01.021

[16] Hagmann P, Cammoun L, Gigandet X, Meuli R, Honey CJ, et al (2008) Mapping the structural core of human cerebral cortex. PLoS Biol 6: e159. 18597554 10.1371/journal.pbio.0060159 PMC2443193

[17] Daducci A, Gerhard S, Griffa A, Lemkaddem A, Cammoun L, et al (2012) The connectome mapper: an open-source processing pipeline to map connectomes with mri. PLoS One 7: e48121. 23272041 10.1371/journal.pone.0048121 PMC3525592

[18] Singh KK, Park KJ, Hong EJ, Kramer BM, Greenberg ME, et al (2008) Developmental axon pruning mediated by BDNF-p75NTR-dependent axon degeneration. Nat Neurosci 11: 649–658. 18382462 10.1038/nn.2114

[19] Cao L, Dhilla A, Mukai J, Blazeski R, Lodovichi C, et al (2007) Genetic modulation of BDNF signaling affects the outcome of axonal competition in vivo. Curr Biol 17: 911–921. 17493809 10.1016/j.cub.2007.04.040 PMC2175069

[20] Gogtay N, Giedd JN, Lusk L, Hayashi KM, Greenstein D, et al (2004) Dynamic mapping of human cortical development during childhood through early adulthood. Proc Natl Acad Sci U S A 101: 8174–8179. 15148381 10.1073/pnas.0402680101 PMC419576

[21] Harris SE, Fox H, Wright AF, Hayward C, Starr JM, et al (2006) The brain-derived neurotrophic factor Val66Met polymorphism is associated with age-related change in reasoning skills. Mol Psychiatry 11: 505–513. 16446742 10.1038/sj.mp.4001799

[22] Gajewski PD, Hengstler JG, Golka K, Falkenstein M, Beste C (2011) The Met-allele of the BDNF Val66Met polymorphism enhances task switching in elderly. Neurobiol Aging 32 2327: e7–2327.19. 10.1016/j.neurobiolaging.2011.06.010 21803453

[23] Mandelman SD, Grigorenko EL (2012) BDNF Val66Met and cognition: all, none, or some? A meta-analysis of the genetic association. Genes Brain Behav 11: 127–136. 21980924 10.1111/j.1601-183X.2011.00738.x PMC3268899

[24] Petryshen TL, Sabeti PC, Aldinger KA, Fry B, Fan JB, et al (2010) Population genetic study of the brain-derived neurotrophic factor (BDNF) gene. Mol Psychiatry 15: 810–815. 19255578 10.1038/mp.2009.24 PMC2888876

[25] Shimizu E, Hashimoto K, Iyo M (2004) Ethnic difference of the BDNF 196G/A (val66met) polymorphism frequencies: the possibility to explain ethnic mental traits. Am J Med Genet B Neuropsychiatr Genet 126B: 122–123. 15048661 10.1002/ajmg.b.20118

[26] Oldfield RC (1971) The assessment and analysis of handedness: the Edinburgh inventory. Neuropsychologia 9: 97–113. 5146491 10.1016/0028-3932(71)90067-4

[27] Buysse DJ, Reynolds C 3rd, Monk TH, Berman SR, Kupfer DJ (1989) The Pittsburgh Sleep Quality Index: a new instrument for psychiatric practice and research. Psychiatry Res 28: 193–213. 2748771 10.1016/0165-1781(89)90047-4

[28] Horne JA, Ostberg O (1976) A self-assessment questionnaire to determine morningness-eveningness in human circadian rhythms. Int J Chronobiol 4: 97–110. 1027738

[29] Roenneberg T, Wirz-Justice A, Merrow M (2003) Life between clocks: daily temporal patterns of human chronotypes. J Biol Rhythms 18: 80–90. 12568247 10.1177/0748730402239679

[30] Johns MW (1991) A new method for measuring daytime sleepiness: the Epworth sleepiness scale. Sleep 14: 540–545. 1798888 10.1093/sleep/14.6.540

[31] Beck AT, Epstein N, Brown G, Steer RA (1988) An inventory for measuring clinical anxiety: psychometric properties. J Consult Clin Psychol 56: 893–897. 3204199 10.1037//0022-006x.56.6.893

[32] Steer RA, Ball R, Ranieri WF, Beck AT (1997) Further evidence for the construct validity of the Beck depression Inventory-II with psychiatric outpatients. Psychol Rep 80: 443–446. 9129364 10.2466/pr0.1997.80.2.443

[33] Raven J, Raven JC, Court JH (1998) Manual for Raven's Progressive Matrices and Vocabulary Scales. Oxford, U.K: Oxford Psychologists Press.

[34] Gorgolewski K, Burns CD, Madison C, Clark D, Halchenko YO, et al (2011) Nipype: a exible, lightweight and extensible neuroimaging data processing framework in python. Front Neuroinform 5: 13. 21897815 10.3389/fninf.2011.00013 PMC3159964

[35] Smith SM, Jenkinson M, Woolrich MW, Beckmann CF, Behrens TEJ, et al (2004) Advances in functional and structural MR image analysis and implementation as FSL. Neuroimage 23 Suppl 1S208–S219. 15501092 10.1016/j.neuroimage.2004.07.051

[36] Tournier JD, Calamante F, Gadian DG, Connelly A (2004) Direct estimation of the fiber orientation density function from diffusion-weighted MRI data using spherical deconvolution. Neuroimage 23: 1176–1185. 15528117 10.1016/j.neuroimage.2004.07.037

[37] Tournier JD, Calamante F, Connelly A (2007) Robust determination of the fibre orientation distribution in diffusion MRI: non-negativity constrained super-resolved spherical deconvolution. Neuroimage 35: 1459–1472. 17379540 10.1016/j.neuroimage.2007.02.016

[38] Smith RE, Tournier JD, Calamante F, Connelly A (2012) Anatomically-constrained tractography: improved diffusion mri streamlines tractography through effective use of anatomical information. Neuroimage 62: 1924–1938. 22705374 10.1016/j.neuroimage.2012.06.005

[39] Smith RE, Tournier JD, Calamante F, Connelly A (2013) Sift: Spherical-deconvolution informed filtering of tractograms. Neuroimage 67: 298–312. 23238430 10.1016/j.neuroimage.2012.11.049

[40] Rubinov M, Sporns O (2010) Complex network measures of brain connectivity: uses and interpretations. Neuroimage 52: 1059–1069. 19819337 10.1016/j.neuroimage.2009.10.003

[41] Hagberg AA, Schult DA, Swart PJ (2008) Exploring network structure, dynamics, and function using NetworkX. In: Proceedings of the 7th Python in Science Conference (SciPy2008). Pasadena, CA USA, 11–15.

[42] Gerhard S, Daducci A, Lemkaddem A, Meuli R, Thiran JP, et al (2011) The connectome viewer toolkit: an open source framework to manage, analyze, and visualize connectomes. Front Neuroin form 5: 3. 10.3389/fninf.2011.00003 PMC3112315 21713110

[43] Rasmussen CE, Williams CKI (2005) Gaussian Processes for Machine Learning (Adaptive Computation and Machine Learning). The MIT Press.

[44] Schrouff J, Rosa MJ, Rondina JM, Marquand AF, Chu C, et al.. (2013) Pronto: Pattern recognition for neuroimaging toolbox. Neuroinformatics. 10.1007/s12021-013-9178-1 PMC3722452 23417655

