# Supplementary figures and images for: Altered White Matter Architecture in BDNF Met Carriers
Source: PLoS One. 2013 Jul 31;8(7):e69290. doi: 10.1371/journal.pone.0069290 (PMC3729843; doi:10.1371/journal.pone.0069290)

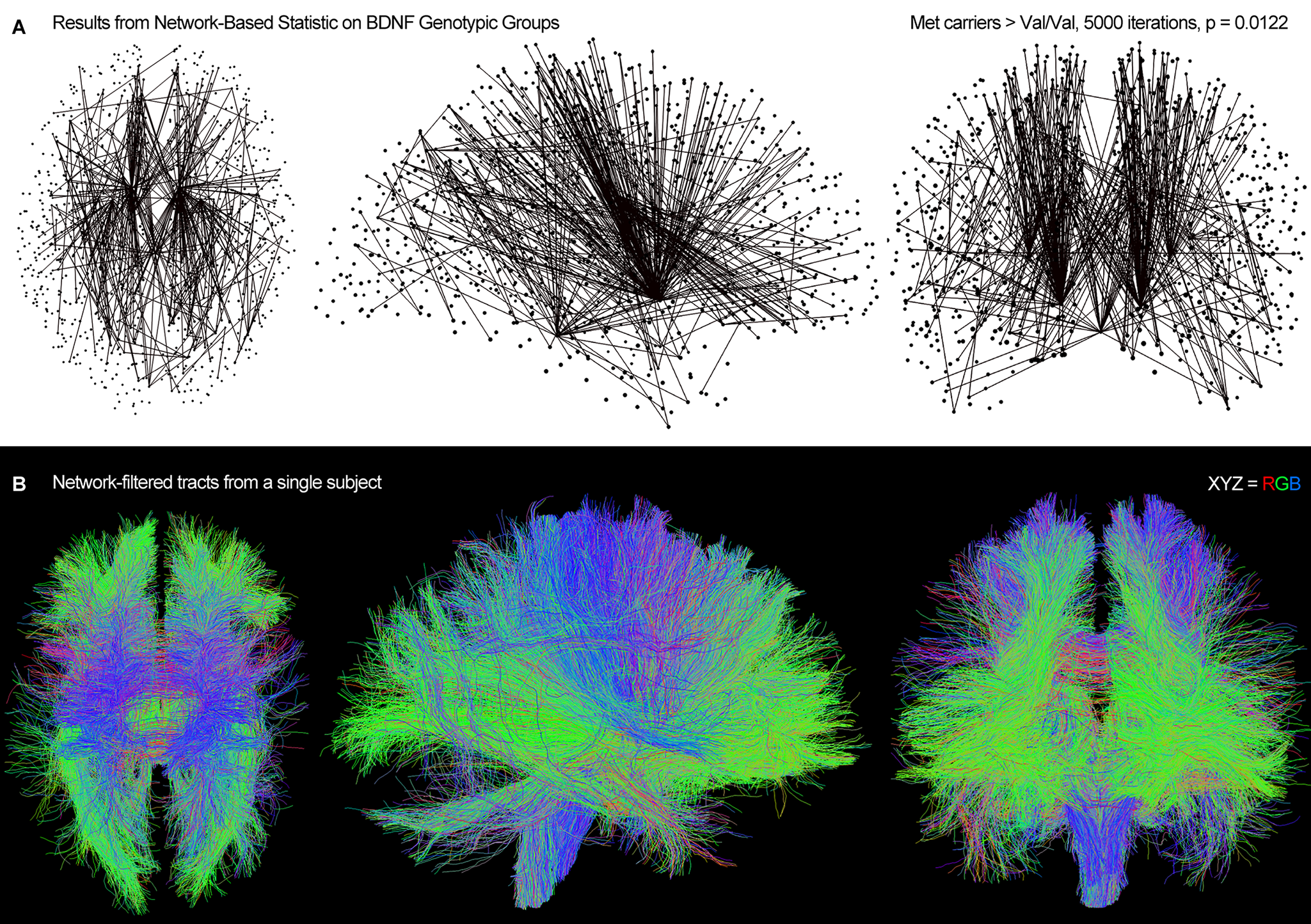

Supplement: Figure S1 — Edge weights are stronger in Met carriers. (a) In the structural component pictured each inter-regional connection has a significantly higher number of tracks for Met carriers. (b) The tracks shown are produced by filtering a single subject's tracts using the connections from the network shown in (a). (TIF) [file pone.0069290.s001.tif]

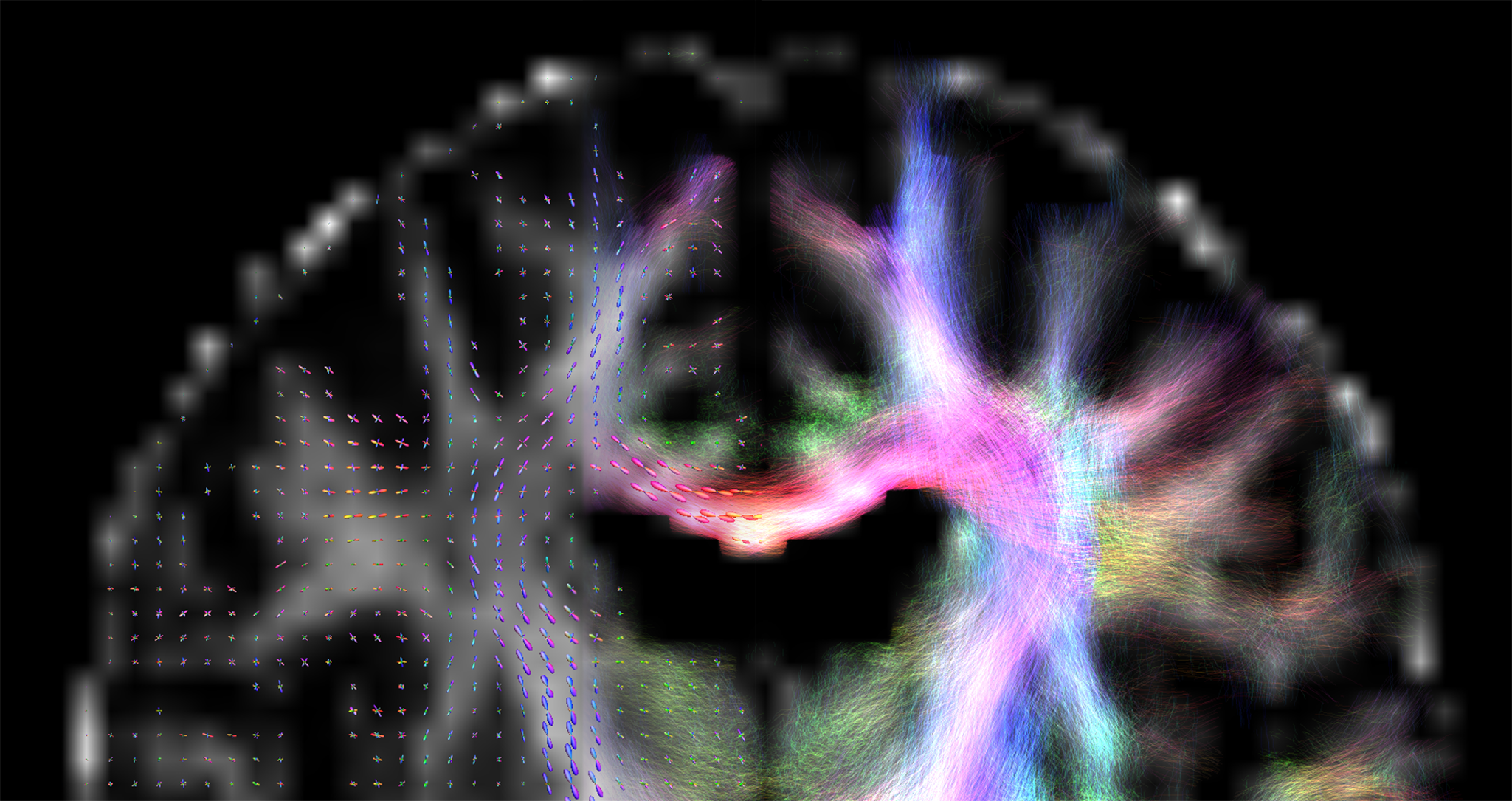

Supplement: Figure S2 — Tracks and Orientation Distribution Functions for a single subject. Combined figure for visualizing the results of the spherical deconvolution and probabilistic fiber tractography steps in the processing pipeline. (TIF) [file pone.0069290.s002.tif]

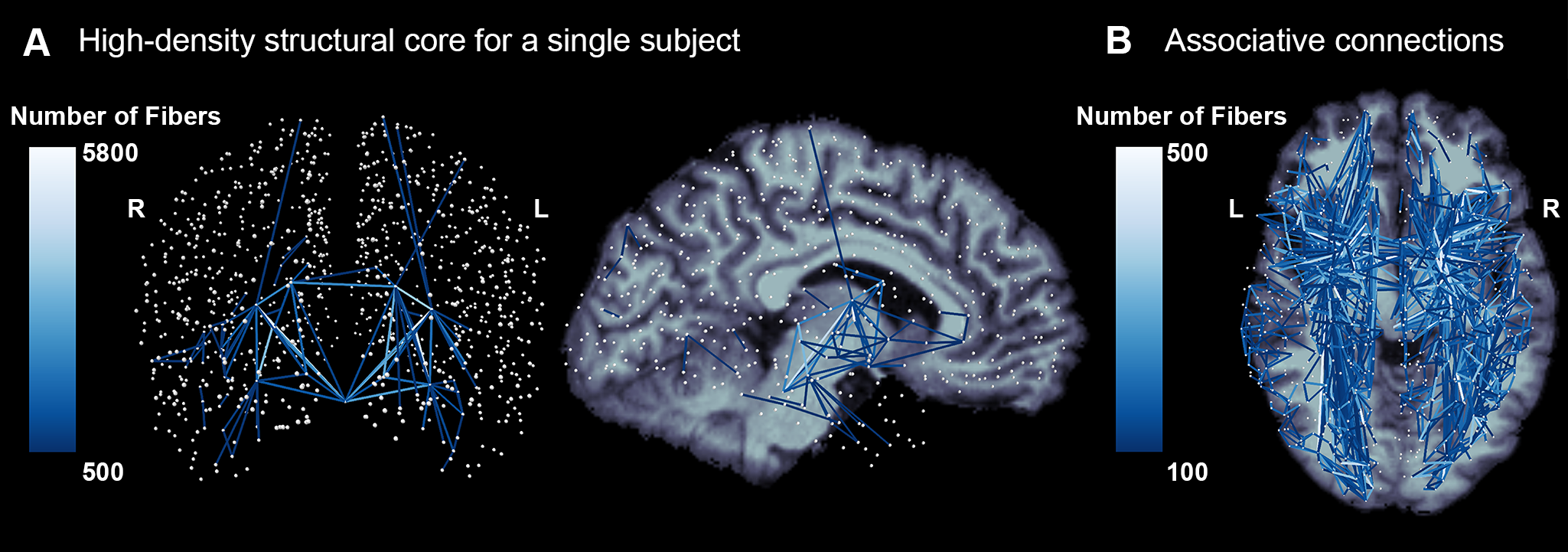

Supplement: Figure S3 — Structural connectome for a single subject. Structural connectivity network built from the Lausanne 2008 regional atlas – with each region displayed as a node – and a set of 300,000 fiber tracks. Colored edge weights represent the number of tracks that provide any connection between any pair of regions. The figure is divided into ranges of edge weights for optimal visualization of the (a) high-valued structural core and the (b) low-valued associative connections. (TIF) [file pone.0069290.s003.tif]

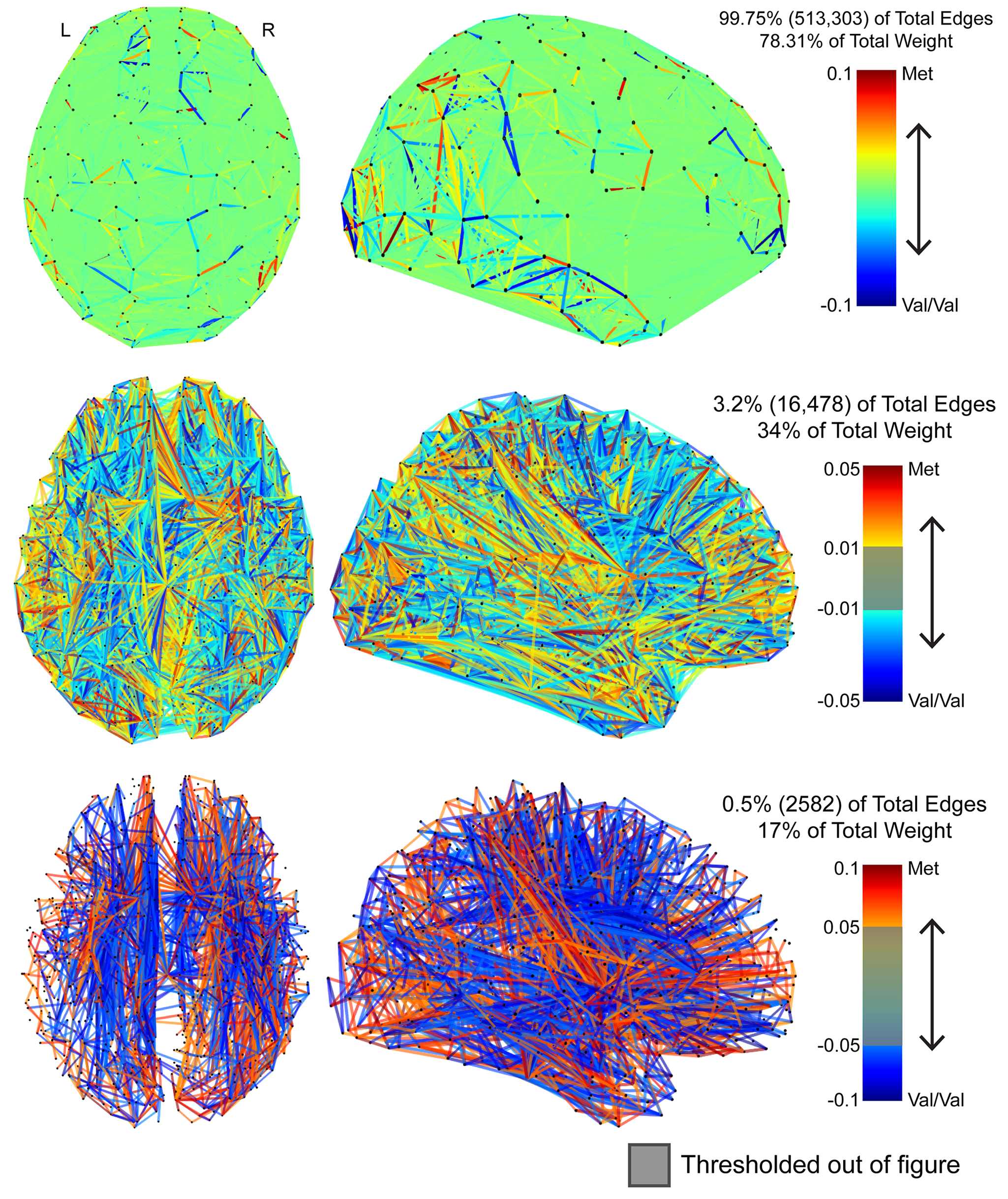

Supplement: Figure S4 — Detailed dissection of the classification weights. (a) The complement of Figure 2 from the main text. This network details the edges that were filtered in the main text figure, and shows 99.75 of the edges, which represent only 78 of the total weight. (b) A set of very low contribution edges between genotypic groups. These very low-valued edges are difficult to interpret. (c) The highest valued edges that were thresholded out of Figure 2 in the main text. A pattern of posterior parietal and medial frontal connectivity can be inferred in the Met carriers, but the abundance of edges is still complex to visualize. (TIF) [file pone.0069290.s004.tif]
