# Supplementary material for: Altered White Matter Architecture in BDNF Met Carriers
Source: PLoS One. 2013 Jul 31;8(7):e69290. doi: 10.1371/journal.pone.0069290 (PMC3729843; doi:10.1371/journal.pone.0069290)
Supplement: File S1 — Table S1– Psychological questionnaire results. Values reflect mean ± standard deviation. Table S2– Connectome edge weights. This table details a single random (Val) subject's network edges. The vast majority of the edges had weights below a fiber count of 100. (PDF) [file pone.0069290.s005.pdf]

Supplementary Information for Altered white matter architecture in BDNF Met carriers

Erik Ziegler[1,*], Ariane Foret[1], Laura Mascetti[1], Vincenzo Muto[1], Anahita Le Bourdiec-Shaffii[1], Johan Stender[1], Evelyne Balteau[1], Vinciane Dideberg[2], Vincent Bours[2], Pierre Maquet[1,3], Christophe Phillips[1,4]
1 Cyclotron Research Centre, Université de Liège, Liège, Belgium, Liège, Liège, Belgium
2 Department of Human Genetics, CHU Sart Tilman, Liège, Belgium
3 Department of Neurology, CHU Liège, Liège, Belgium
4 Department of Electrical Engineering and Computer Science, University of Liège, Belgium
∗ **E-mail: erik.ziegler@ulg.ac.be**

The following equations and figures are supplementary material.

The networks in our study have 1015 anatomically defined nodes. This leads to:

$$\begin{aligned} N_{edges} &= \frac{N_{nodes} * (N_{nodes} - 1)}{2} \\ N_{edges} &= \frac{1015 * (1015 - 1)}{2} \\ N_{edges} &= 514,605 \end{aligned} \tag{1}$$

The total of all the classifier weights was obtained by summing the absolute value of all the weights:

$$\begin{aligned} W_{total} &= \sum_{i=0,j=0}^{N_{nodes}} |W_{i,j}| \\ &= 2077.6096558590079 \end{aligned} \tag{2}$$

For each of the thresholding windows we calculated the percent of total weight represented. For example, in Figure 2 of the main text, we calculated the weight and amount of edges using the following method. First, we defined a binarizing threshold function to obtain the number of edges:

$$f(x) = \begin{cases} 1 & x \text{ if -0.1} \leq n \leq 0.1 \\ 0 & x \text{ if } n > 0.1 \text{ or } n < \text{-0.1} \end{cases} \tag{3}$$

Next, we used this to obtain the total number of thresholded edges, and their percent of the total edges.

$$N_{edges}^{thresh} = \sum_{i=0,j=0}^{N_{nodes}} f(W_{i,j}) \tag{4}$$

The percent of edges that remain are simply:

$$\begin{aligned} Percent_{edges}^{thresh} &= \frac{N_{edges}^{thresh}}{N_{edges}} * 100 \\ Percent_{edges}^{thresh} &= \frac{1302}{514605} \\ Percent_{edges}^{thresh} &= 0.25\% \end{aligned} \tag{5}$$

The total weight of the edges that are within the threshold regions can be obtained similarly:

$$W_{total}^{thresh} = \sum_{i=0,j=0}^{N_{nodes}} f(W_{i,j}) * |W_{i,j}| \tag{6}$$

The percent of the total classifier weight contained within the thresholded edges is therefore:

$$
\begin{aligned}
Percent_{weight}^{thresh} &= \frac{W_{total}^{thresh}}{W_{total}} * 100 \qquad (7)\\
Percent_{weight}^{thresh} &= \frac{450.57456206441788}{2077.6096558590079}\\
Percent_{weight}^{thresh} &= 21\%
\end{aligned}
$$

For future studies it may be simpler to only consider edges that exist in at least one structural network. This will speed classification and make visualization easier. That is to say, the classification should be given a mask that contains only edges that exist in the union of all all subject's networks. This can be expressed mathematically with set theory as:

$$Mask_{inclusive} = Subj_1 \cup Subj_2 \cup \cdots Subj_n \qquad (8)$$

Figure Legends

Figure 1. Edge weights are stronger in Met carriers. (**a**) In the structural component pictured each inter-regional connection has a significantly higher number of tracks for Met carriers. (**b**) The tracks shown are produced by filtering a single subject's tracts using the connections from the network shown in (**a**).

Figure 2. Tracks and Orientation Distribution Functions for a single subject. Combined figure for visualizing the results of the spherical deconvolution and probabilistic fiber tractography steps in the processing pipeline.

Figure 3. Structural connectome for a single subject. Structural connectivity network built from the Lausanne2008 regional atlas - with each region displayed as a node - and a set of 300,000 fiber tracks. Colored edge weights represent the number of tracks that provide any connection between any pair of regions. The figure is divided into ranges of edge weights for optimal visualization of the (**a**) high-valued structural core and the (**b**) low-valued associative connections.

Tables

Figure 4. Detailed dissection of the classification weights. (**a**) The complement of Figure 2 from the main text. This network details the edges that were filtered in the main text figure, and shows 99.75% of the edges, which represent only 78% of the total weight. (**b**) A set of very low contribution edges between genotypic groups. These very low-valued edges are difficult to interpret. (**c**) The highest valued edges that were thresholded out of Figure 2 in the main text. A pattern of posterior parietal and medial frontal connectivity can be inferred in the Met carriers, but the abundance of edges is still complex to visualize.

Table 1. Psychological questionnaire results

Measure	Val/Val	Met carriers	t-Test p-Value	Meaning
Age	21.4 ± 1.7	20.4 ± 1.3	0.07	
IQ	55.6 ± 2.5	56.6 ± 2.6	0.26	
Timed IQ	25.2 ± 5.9	27.1 ± 9.6	0.51	
Body Mass Index (BMI)	22.3 ± 1.9	21.4 ± 2.1	0.21	
Beck Anxiety Inventory (BAI)	4.1 ± 2.7	3.9 ± 2.7	0.82	Normal
Beck Depression Inventory (BDI-II)	3.5 ± 4	1.8 ± 2.3	0.14	Normal
Pittsburgh Sleep Quality Index (PSQI)	2.9 ± 1.3	2.9 ± 1.1	0.98	Good Sleep
Horne-Osberg Chronotype (HO)	53.4 ± 6.5	56.1 ± 7.6	0.27	Neutral Chronotype
Munich Chronotype	4.3 ± 0.7	4.1 ± 0.4	0.58	Normal
Epworth Sleepiness Scale	5.2 ± 2.3	5.8 ± 3.4	0.59	Normal

Values reflect mean ± standard deviation

Table 2. Connectome edge weights

Measure	Value
Number of Edges	65,785
Graph density	12.78%
Minimum Edge Weight	1
Maximum Edge Weight	4737
Total Edge Weight	802,470
Mean Edge Weight	12.2
Standard Deviation in Edge Weight	55.8
Percent of Edges with Weight = 1	41.2%
Percent of Edges with Weight = 2	14.6%
Percent of Edges with Weight = 3	8.1%
Percent of Edges with Weight <= 5	72.9%
Percent of Edges with Weight < 100	97.6%
Percent of Edges with Weight >= 100	2.4%

This table details a single random (Val) subject's network edges. The vast majority of the edges had weights below a fiber count of 100.
